# Epigenetic Landscape of Liquid Biopsy in Colorectal Cancer

**DOI:** 10.3389/fcell.2021.622459

**Published:** 2021-02-05

**Authors:** Aitor Rodriguez-Casanova, Nicolás Costa-Fraga, Aida Bao-Caamano, Rafael López-López, Laura Muinelo-Romay, Angel Diaz-Lagares

**Affiliations:** ^1^Cancer Epigenomics Laboratory, Translational Medical Oncology Group (Oncomet), Health Research Institute of Santiago (IDIS), University Clinical Hospital of Santiago (CHUS/SERGAS), Santiago de Compostela, Spain; ^2^Roche-Chus Joint Unit, Translational Medical Oncology Group (Oncomet), Health Research Institute of Santiago (IDIS), Santiago de Compostela, Spain; ^3^Translational Medical Oncology Group (Oncomet), Health Research Institute of Santiago (IDIS), University Clinical Hospital of Santiago (CHUS/SERGAS), Santiago de Compostela, Spain; ^4^Centro de Investigación Biomédica en Red Cáncer (CIBERONC), Madrid, Spain; ^5^Liquid Biopsy Analysis Unit, Translational Medical Oncology Group (Oncomet), Health Research Institute of Santiago (IDIS), University Clinical Hospital of Santiago (CHUS/SERGAS), Santiago de Compostela, Spain

**Keywords:** epigenetics, liquid biopsy, biomarkers, colorectal cancer, precision oncology, circulating nucleic acids, CTCs, extracellular vesicles

## Abstract

Colorectal cancer (CRC) is one of the most common malignancies and is a major cause of cancer-related deaths worldwide. Thus, there is a clinical need to improve early detection of CRC and personalize therapy for patients with this disease. In the era of precision oncology, liquid biopsy has emerged as a major approach to characterize the circulating tumor elements present in body fluids, including cell-free DNA and RNA, circulating tumor cells, and extracellular vesicles. This non-invasive tool has allowed the identification of relevant molecular alterations in CRC patients, including some indicating the disruption of epigenetic mechanisms. Epigenetic alterations found in solid and liquid biopsies have shown great utility as biomarkers for early detection, prognosis, monitoring, and evaluation of therapeutic response in CRC patients. Here, we summarize current knowledge of the most relevant epigenetic mechanisms associated with cancer development and progression, and the implications of their deregulation in cancer cells and liquid biopsy of CRC patients. In particular, we describe the methodologies used to analyze these epigenetic alterations in circulating tumor material, and we focus on the clinical utility of epigenetic marks in liquid biopsy as tumor biomarkers for CRC patients. We also discuss the great challenges and emerging opportunities of this field for the diagnosis and personalized management of CRC patients.

## Introduction

Colorectal cancer (CRC) is the third most frequently detected cancer in both sexes worldwide. In particular, this tumor type accounts for ~10% of all diagnosed cancer cases, with ~1.8 million new cases estimated in 2018. Importantly, CRC is the second leading cause of cancer mortality in the world, being responsible for ~9% of all cancer deaths (Bray et al., 2018). Surgery remains the most common treatment for non-metastatic CRC, while the administration of adjuvant chemotherapy is mainly restricted to stage III tumors. Importantly, most CRC patients are diagnosed at an advanced stage because symptoms normally appear after disease progression (John et al., 2011). The 5-year survival rate after surgery of localized CRC patients is over 90%, while patients affected by stage III and IV tumors, exhibiting local lymph node invasion or distant metastases, respectively, normally show poor overall survival rates (Mattiuzzi et al., 2019). The high incidence and mortality of CRC highlight the clinical need for novel strategies to improve early CRC detection and personalize the management of patients with this type of tumor.

Currently, various screening assays are being used to detect CRC at an early stage. These detection strategies include the fecal immunochemical test (FIT), a non-invasive and cost-effective assay for detecting the presence of fecal hemoglobin (Song and Li, 2016). A positive result of this test implies the recommendation of a colonoscopy, which is the gold standard diagnostic technique for CRC detection. However, this method is clearly invasive, requires considerable patient preparation, and can eventually lead to serious complications (Triantafillidis et al., 2015).

Importantly, a refined understanding of the molecular aspects of CRC has recently been achieved owing to the application of next generation sequencing (NGS)-based approaches, which revealed a wide intratumor heterogeneity and general genomic instability (Molinari et al., 2018). Based on the advances in the molecular characterization of CRC, new biological drugs targeting vascular endothelial growth factors (VEGFRs), such as bevacizumab (Rosen et al., 2017), or epidermal growth factors (EGFRs), including cetuximab and panitumumab (Qin et al., 2018; Taniguchi et al., 2020), have improved the survival of patients, mainly in the context of metastatic CRC (mCRC). In addition, promising targeted therapies for mCRC are being evaluated in pre-clinical and clinical studies, including new drugs directed against different components of EGF/EGFR, VEGF/VEGFR, and HGF/c-MET pathways (Xie Y. H. et al., 2020). On the other hand, several recent pre-clinical studies have shown that blocking the PD-1/PD-L1 interaction or CTLA-4 with immune checkpoint inhibitors prevents colorectal tumor cells to escape from immune surveillance (Fiegle et al., 2019; Zhang Y. et al., 2020). In this sense, the use of immune checkpoint inhibitors has provided good outcomes in the treatment of mCRC with microsatellite instability (MSI) (Overman et al., 2018), which can be detected in ~15% of CRC patients (Ward et al., 2001; Le et al., 2015). Importantly, several molecular biomarkers, such as the presence of *KRAS/NRAS/BRAF* mutations and MSI markers, have been approved for supporting the selection of targeted therapies (Lo Nigro et al., 2016). However, these predictive biomarkers are currently analyzed in tumor tissue samples, which are not always available during the disease evolution and can provide partial information of the molecular profile of colorectal tumors, mainly in the metastatic setting.

Liquid biopsy has emerged in recent years as an important approach to address and overcome such limitations. Indeed, this non-invasive strategy allows to observe the molecular landscape of circulating tumor elements in body fluids to obtain diagnostic, prognostic, and therapy response biomarkers that improve the management of cancer patients (Siravegna et al., 2017). The analysis of these liquid biopsy components in several body fluids of CRC patients has highlighted relevant molecular alterations, such as those depending on epigenetic mechanisms (Lofton-Day et al., 2008; Maminezhad et al., 2020). Importantly, the combination of liquid biopsy and detection of epigenetic alterations represents a great opportunity in cancer research for the identification of new non-invasive clinical biomarkers to improve the detection of CRC and personalize the management of this disease.

In this review, we provide an overview of the epigenetic landscape of liquid biopsies in CRC. We describe the concept and clinical application of liquid biopsy, the most relevant epigenetic mechanisms (DNA modifications, histone modifications and nucleosome positioning, and non-coding RNAs), and the implications of their deregulation in cancer cells and liquid biopsy. Furthermore, we summarize the methodologies used for detecting these epigenetic modifications in liquid biopsy, and describe the clinical utility of epigenetic marks in liquid biopsy as tumor biomarkers for CRC patients. Finally, we discuss the great challenges and opportunities of liquid biopsy epigenetics for the detection of CRC and management of CRC patients.

## Liquid Biopsy

In the era of precision oncology, liquid biopsies represent a key element for cancer detection, to guide treatment selection and monitor tumor evolution in real time. A liquid biopsy consists of any body fluid that contains tumor material suitable for molecular characterization. Therefore, this term includes blood, the most used human liquid sample, but also other fluids such as urine, ascitic fluid, pleural effusion, cerebrospinal fluid, and saliva. Both primary tumors and metastases can release tumor material into these body fluids, mainly consisting of circulating tumor cells (CTCs), nucleic acids (cNAs), and extracellular vesicles (cEVs). These circulating elements constitute a valuable source of non-invasive biomarkers and information about the molecular mechanisms underpinning tumor dissemination and evolution (Siravegna et al., 2017).

Although Thomas Ashworth described the presence of tumor cells in the blood of breast cancer patients for the first time in 1869 (Ashworth, 1869), it was not until recent years that the scientific community focused their attention on the study of blood CTCs. Like most circulating tumor elements, CTCs are poorly concentrated in blood, and thus require the implementation of highly sensitive and specific strategies for their enrichment and subsequent detection. Initially, the most common strategy to isolate CTCs was immune enrichment, based on the presence of cell surface markers such as the epithelial cell adhesion molecule (EpCAM) (Allard et al., 2004). For example, the Food and Drug Administration (FDA)-approved CellSearch System can isolate EpCAM-positive cells and determine the number of CTCs in a sample after an immunofluorescence assay to detect epithelial and hematopoietic markers (Cristofanilli et al., 2004). This platform has been employed in numerous studies to quantify CTCs in CRC patients, demonstrating the clinical value of CTC enumeration as a prognostic and follow-up biomarker (Cohen et al., 2008; Sastre et al., 2012; Bork et al., 2015). However, this strategy has not been widely adopted as a clinical tool, mainly due to the lack of clear benefits in terms of accuracy of treatment decisions. Nevertheless, the technology for isolating and characterizing CTCs has continued to improve during the last decade, mainly through the application of antigen-independent technologies allowing to isolate a broader and more molecularly heterogeneous CTC population (Barbazan et al., 2017). Despite the difficulty of translating CTCs into the clinical context, their molecular characterization has provided valuable information for understanding how colorectal tumor cells are able to disseminate, implant at distant locations, and generate metastasis (Barbazan et al., 2012, 2017). Such molecular characterization, including epigenetic mechanisms, is vital for unraveling the biological aspects of CRC and identifying new clinical and therapeutic biomarkers to manage this disease.

In contrast to the limited translation of CTCs into routine clinical practice, the analysis of cNAs has started to be applied to oncologic therapy selection. These analyses mainly focus on circulating cell-free DNA (cfDNA), released as a consequence of cell death and characterized by a high fragmentation in biological fluids (size, 160–180 bp). cfDNA generally contains small fractions of circulating tumor DNA (ctDNA), in a range as low as 0.01–1% in patients with advanced tumors (Thierry et al., 2016). Of note, several genetic alterations, such as point mutations, copy number variations, small indels, and translocations, together with epigenetic modifications, can be studied in cfDNA from different body fluids in a non-invasive and comprehensive way (Bardelli and Pantel, 2017). Furthermore, in 2014 Bettegowda et al. demonstrated that ctDNA is detectable in most patients with mCRC and in a considerable percentage of non-metastatic patients (Bettegowda et al., 2014). These results provided the basis for numerous studies that have demonstrated that it is possible to characterize the molecular alterations of CRC with high accuracy, thereby predicting the information obtained by standard image-based follow-up. For example, there is clear scientific evidence of the robustness of ctDNA analyses to monitor RAS mutational status without the need for invasive tissue biopsies. This approach is also interesting for improving the selection of anti-EGFR therapy at different time points of disease evolution (Siravegna et al., 2015; Vidal et al., 2017). Moreover, recent technological improvements have favored the application of ctDNA analysis in patients with early CRC as well as for the detection of minimal residual disease (MRD) after surgery (Cohen et al., 2018).

Among cNAs, numerous non-coding RNAs (ncRNAs) can be detected in liquid biopsy. Several studies have highlighted the role of ncRNAs in cell-to-cell communication through the promotion of differential gene expression in tumor cells and stroma, which has a relevant impact on cancer progression and therapy resistance (Anfossi et al., 2018; Pardini et al., 2019). Although cNA-based applications for CRC are still in the clinical validation phase, current genetic and epigenetic data are promising and will soon support a more extensive use of cNA analysis to manage this type of cancer.

Other circulating elements of great interest found in liquid biopsy are cEVs. These vesicles are a complex population of cell-derived membranous structures released by cells through different mechanisms, which can be grouped into exosomes (ranging from 30 to 100 nm), microvesicles (50–2,000 nm), and apoptotic bodies (500–4,000 nm) (Akers et al., 2013; van Niel et al., 2018). These vesicles play an important role in cancer, mediating the interaction between tumor and stromal cells, promoting cell proliferation and invasion, and significantly contributing to the establishment of pre-metastatic niches (de la Fuente et al., 2015; Becker et al., 2016). Such functions are mediated by their membrane components, but also their molecular cargo, composed of proteins, messenger RNAs (mRNAs), ncRNAs, and single- or double-stranded DNA (van Niel et al., 2018). Importantly, cEVs can be found in different body fluids at high concentrations. Their isolation is mainly achieved by ultracentrifugation, immunoaffinity, or precipitation strategies (Bu et al., 2019; Jayaseelan, 2020), and the selection of the optimal isolation strategy is a crucial point, significantly influencing the characteristics of the isolated vesicles. The presence of relevant molecular alterations has been described in exosomal DNA from cancer patients (Hao et al., 2017; Castellanos-Rizaldos et al., 2018). Furthermore, exosomal ncRNAs obtained from serum and plasma have also been explored in connection with CRC, with promising results (Matsumura et al., 2015).

In summary, the study of cNAs, CTCs, and cEVs has shown significant potential for early CRC diagnosis, therapy selection, and disease monitoring, through the analysis of several molecular alterations such as those depending on various epigenetic mechanisms, which are examined in detail in the present review.

## The Epigenetic Machinery and Cancer

**Figure 1:**
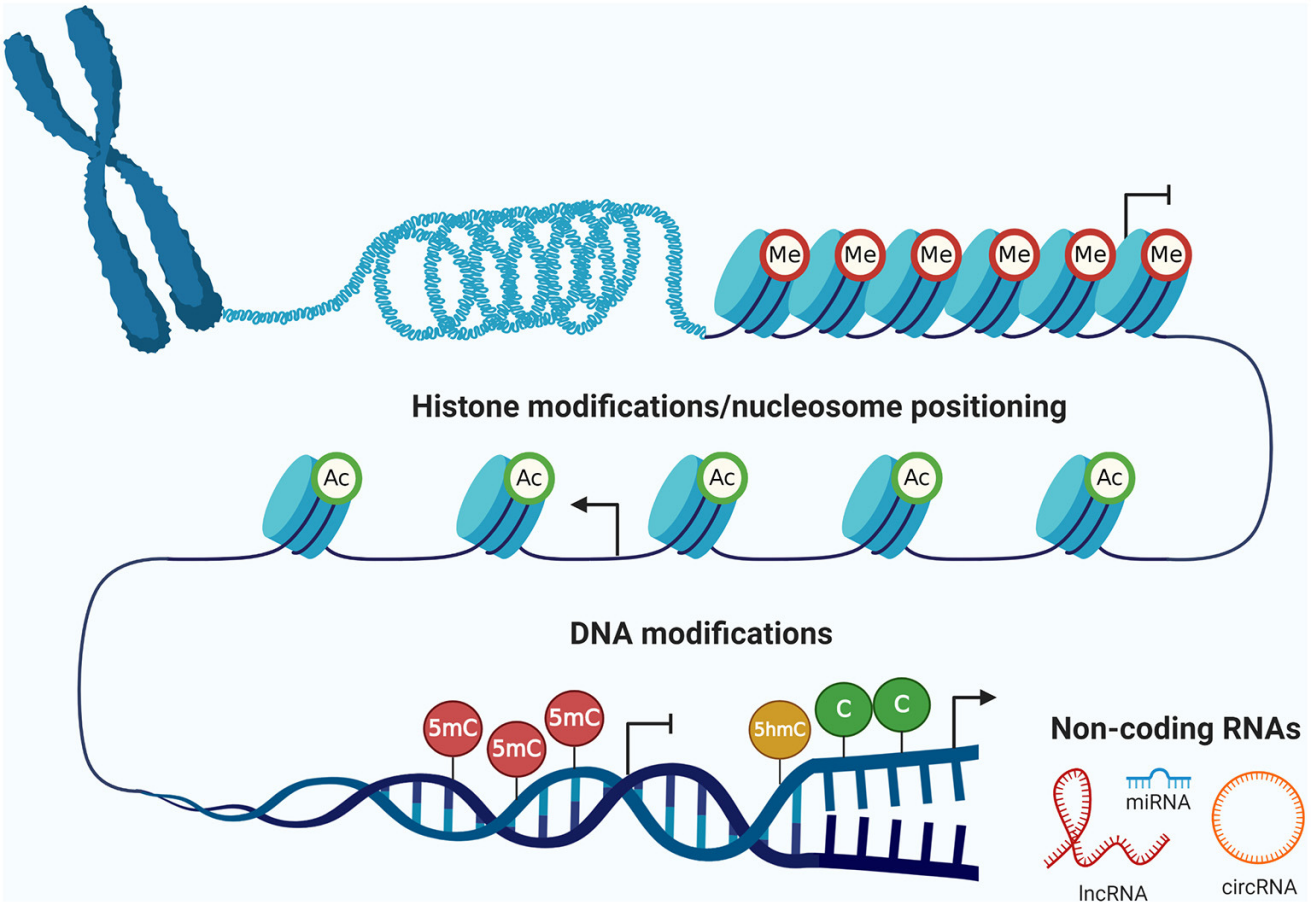
The epigenetic machinery. There are three fundamental epigenetic mechanisms, represented by (i) DNA modifications (methylation and hydroxymethylation), (ii) post-translational modifications of histones and nucleosome positioning, and (iii) non-coding RNAs. These epigenetic layers are highly interrelated among them and regulate gene expression of relevant biological processes in normal cells. However, these mechanisms can be deregulated in tumor cells leading to cancer development and progression. Me, methylation of histones; Ac, acetylation of histones; 5mC, 5-methylcytosine; 5hmC, 5-hydroxymethylcytosine; C, cytosine; lncRNA, long non-coding RNA; miRNA, microRNA; circRNA, circular RNA. Created with BioRender.com.

The concept of epigenetics was postulated for the first time in 1942 by Waddington (2012), and can now be defined as the study of hereditary changes in the activity and expression of genes that take place without alterations of the DNA sequence (Holliday, 1987; Berger et al., 2009). The epigenetic machinery has several mechanisms (Figure 1), including DNA methylation and hydroxymethylation, histone modifications and nucleosomes positioning, and ncRNAs (Rodriguez-Paredes and Esteller, 2011). These mechanisms play an important role in regulating gene expression during many biological processes, such as embryonic development, imprinting, and tissue differentiation (Sharma et al., 2010). However, the deregulation of all these epigenetic layers has important implications for cancer development and progression (Mari-Alexandre et al., 2017).

### DNA Modifications: Methylation and Hydroxymethylation

DNA methylation is the most widely described epigenetic mechanism. This covalent modification of DNA consists in the incorporation of a methyl group (CH_3_) to the 5′ carbon of cytosines in cytosine-phosphate-guanine (CpG) dinucleotides to produce 5-methylcytosine (5mC) (Portela and Esteller, 2010). Such incorporation is regulated by DNA methyltransferases (DNMTs), including DNMT1, DNMT3A, and DNMT3B, which catalyze the transfer of methyl groups from S-adenosyl-L-methionine (SAM) to cytosines. In particular, DNMT3A and DNMT3B participate in producing *de novo* methylation patterns in cells, while DNMT1 maintains the parental methylation profile in each cell division (Gowher and Jeltsch, 2002; Jaenisch and Bird, 2003). DNA methylation usually occurs in specific regions of the genome with high density of CpG dinucleotides, called CpG islands (CGIs). These CpG-enriched sequences are usually located in the promoter regions of genes but can also be present in intragenic regions, including gene bodies (Diaz-Lagares et al., 2016a; Arechederra et al., 2018). According to their distance from CGIs, genomic sequences can be defined as CGI shores (up to 2 kb from CGIs), shelves (2–4 kb from CGIs), and open sea (>4 kb from CGIs) (Qu et al., 2014). In addition, DNA methylation can affect not only the intragenic but also the intergenic regions of the genome, increasing the complexity of this layer of epigenetic regulation (Zhao S. G. et al., 2020).

DNA methylation plays an important role in regulating gene expression as well as in maintaining the integrity and conformation of DNA, thereby protecting it from the potential damage by mobile genetic elements (Herceg and Vaissiere, 2011). This epigenetic modification can be enhanced (hypermethylation) or suppressed (hypomethylation) in different regions of the genome (Portela and Esteller, 2010). In cancer, hypermethylation of CGIs in promoters is usually linked to silencing of both coding and non-coding tumor suppressors (Diaz-Lagares et al., 2016a,b). However, hypomethylation of CpG-poor regions has been associated with proto-oncogene expression, genomic instability, and malignant transformation of tumors (Esteller, 2008; Sheaffer et al., 2016).

In human cells, DNA methylation can be reversed by TET (ten-eleven translocation) enzymes, which induce the oxidation of 5mC to 5-hydroxymethylcytosine (5hmC) in a process defined as DNA hydroxymethylation (Tahiliani et al., 2009). This demethylation mechanism can modulate gene expression by adjusting the methylation levels (Xu and Gao, 2020). In cancer, deregulation of TET enzymes can alter the balance of genomic 5mC/5hmC levels, inducing cancer transformation (Chen et al., 2017). In addition, DNA methylation can be reversed through epigenetic-based drugs (epidrugs), which can decrease the methylation levels of hypermethylated genes (Berdasco and Esteller, 2019). For example, 5-azacytidine (5-AZA-CR) and 5-aza-2′-deoxycytidine (5-AZA-CdR, decitabine) are nucleoside analogs approved by the FDA, acting as DNA methyltransferase inhibitors (DNMTi) (Quintas-Cardama et al., 2010).

### Histone Modifications and Nucleosome Positioning

Nucleosomes were first described in 1974 by Kornberg (1974) and represent the basic functional units of chromatin. They are composed of 147 bp of DNA wrapped around an octamer consisting of two copies of four core histone proteins (H2A, H2B, H3, and H4) (Kouzarides, 2007). This core of histones is organized into two H2A–H2B dimers and one H3–H4 tetramer. In addition, nucleosomes bind the linker histone H1, which protects the ~20–50 bp of free DNA (linker DNA) located between neighboring nucleosomal particles (Portela and Esteller, 2010). Nucleosomal histones can undergo different types of reversible post-translational modifications (PTMs), such as acetylation, methylation, phosphorylation, ubiquitylation, and sumoylation, that mainly occur in the histone tail and are enzymatically regulated (Kouzarides, 2007). Importantly, histone PTMs have demonstrated to play a relevant role in normal development and pathogenesis associated with transcriptional regulation, DNA repair and replication, or chromatin condensation (Bannister and Kouzarides, 2011; Bates, 2020). Specifically, multiple histone PTMs related to gene activation or silencing have been described, in particular histone acetylation and methylation. The balance of these types of PTMs is regulated by histone acetyltransferases (HATs), histone deacetylases (HDACs), histone methyltransferases (HMTs), and histone demethylases (HDMs). The possible combinations of PTMs provide various histone modification patterns that have been proposed to constitute a “histone code” associated with open/closed states of chromatin and gene regulation (Strahl and Allis, 2000; Rando, 2012).

In addition to PTMs, the positioning of nucleosomes has shown to be importantly involved in the regulation of chromatin accessibility. Indeed, variations in the position of nucleosomes modulate the binding of RNA polymerase or transcription factors to regulatory elements that control gene expression (Kurup et al., 2019; Huertas et al., 2020). Nucleosome positioning is controlled by chromatin-remodeling proteins (called movers) that shift nucleosomes and allow gene expression (Bates, 2020). Active genes usually present nucleosome-depleted regions (NDR) around their transcription start sites (TSS) that facilitate the accessibility of transcription regulatory proteins (Mavrich et al., 2008). The organization of nucleosomes is dynamically regulated and their turnover in active promoters and enhancers is higher than in inactive regions (Deal et al., 2010; Klemm et al., 2019). Interestingly, nucleosome positioning not only regulates gene expression but also influences the type of DNA fragmentation that can occur in different cellular processes, such as apoptosis, resulting in a non-random fragmentation of DNA (Matassov et al., 2004; Ivanov et al., 2015).

In cancer, epigenetic mechanisms such as nucleosome positioning and PTMs of histones, and/or the enzymes that regulate these modifications, are often deregulated (Hesson et al., 2014; Yang et al., 2014). For example, mutations in histone-modifying enzymes can lead to cancer development due to alterations in the PTM balance of histones (Morin et al., 2010). However, the alteration of histone modifications can be reversed in some types of tumors through the administration of certain epidrugs approved by the FDA (Hoy, 2020).

### Non-coding RNAs

Non-coding transcripts are important regulatory molecules that represent the vast majority of the transcriptome (~98%) (Kapranov et al., 2007). The number of identified ncRNAs has been rapidly increasing in recent years, and currently multiple types of ncRNAs build up the non-coding transcriptome. Similar to other epigenetic mechanisms, ncRNAs have relevant functions in controlling gene expression (Dragomir et al., 2018). Non-coding transcripts are usually classified based on their nucleotide length. Using this criterion, ncRNAs can be divided into: (i) small ncRNAs (sncRNAs), which are shorter than 200 nt and include microRNAs (miRNAs), small interfering RNAs (siRNAs), and piwi-interacting RNAs (piRNAs); and (ii) long ncRNAs (lncRNAs), which are longer than 200 nt and encompass long intergenic ncRNAs (lincRNAs) and long intronic ncRNAs (intronic lncRNAs) (Taft et al., 2010; Esteller, 2011; Memczak et al., 2013). Of note, some ncRNAs have variable lengths and might be attributed to both classes at the same time. This is the case of enhancer RNAs (eRNAs), which originate from transcriptional enhancers, and circular RNAs (circRNAs), which are circularization products arising from splicing events (Leveille et al., 2015; Zhang et al., 2019).

Among sncRNAs, miRNAs have been the most widely described ncRNAs. miRNAs are single-stranded molecules of 18–25 nt that bind to specific regions of target mRNAs, mediating post-transcriptional gene silencing by two possible mechanisms: blocking transcription or triggering mRNA degradation (Lee and Calin, 2011). As a consequence, a single miRNA can control the expression of hundreds of genes, regulating key pathways for cancer tumorigenesis and progression (Garzon et al., 2009). Importantly, miRNAs can have a dual effect in cancer, functioning either as tumor suppressors or oncogenes (oncomiRs) (Volinia et al., 2006; Bayraktar et al., 2017). In addition, different types of cancers have shown specific miRNA signatures, defining the molecular characteristics of tumors (Lu et al., 2005).

Although miRNAs are the most studied ncRNAs, lncRNAs have recently been shown to represent the vast majority of non-coding transcripts (Hon et al., 2017). LncRNAs do not have the potential to encode proteins, but they may exhibit some mRNA-like properties, such as multiexonic gene structures, polyadenylation, presence of 5′ caps, and transcription by RNA polymerase II (Guttman et al., 2009; Derrien et al., 2012). LncRNAs have relevant regulatory functions in the process of gene expression, for example during transcriptional regulation and splicing (Kotake et al., 2011; Leveille et al., 2015). Nevertheless, currently few lncRNAs have been well-characterized, although several studies have shown their implications in cancer, where they act by suppressing (Leveille et al., 2015; Diaz-Lagares et al., 2016a) or promoting the tumoral process (Gupta et al., 2010; Gutschner et al., 2013).

### Interplay Between Epigenetic Mechanisms

In normal mammalian cells, epigenetic modifications can regulate each other through the interplay among distinct epigenetic players, forming a complex regulatory network (Caley et al., 2010; Rose and Klose, 2014). However, this crosstalk is also evident in cancer, where specific methylation and histone modification patterns regulate the expression of different types of ncRNAs, including miRNAs and lncRNAs (Shin et al., 2009; Leveille et al., 2015). Conversely, the disruption of ncRNA expression in cancer cells may alter histone PTMs and DNA methylation levels (Gupta et al., 2010). These are only some examples of the important crosstalk that usually occurs among the different components of the epigenetic machinery.

## Methods for Detecting Epigenetic Marks in Liquid Biopsy

Multiple methodologies have been described at genome-wide or locus-specific level for the analysis of the status of different types of epigenetic marks (Bao-Caamano et al., 2020). However, it is important to consider the advantages and limitations of each method in order to choose the most appropriate approach depending on the type of epigenetic mechanism under investigation and on the conditions of the assay (Kurdyukov and Bullock, 2016). Table 1 shows the most representative methods used for detecting epigenetic marks in liquid biopsy.

**Table 1:** Methods used for the analysis of epigenetic marks in liquid biopsy.

**Feature**	**Approach**	**Method**	**Characteristics of the analysis**	**References**
Methylation	LS	MSP	CpG sites by PCR	Herman et al., 1996
		qMSP	CpG sites by qPCR	Danese et al., 2013
		Methylight	CpG sites with fluorescent probes by qPCR	Eads et al., 2000
		MS-HRM	Regions with CpGs by qPCR and melting curves	Yang et al., 2015
		Multiplexed-scAEBs	CpGs sites in multiple loci by Sanger sequencing	Pixberg et al., 2017
		Methyl-BEAMing	CpG sites by emulsion dPCR	Li et al., 2009
		ddPCR	CpG sites by ddPCR	Picardo et al., 2019
		MBD-ddPCR	Immunoprecitation and ddPCR	Shinjo et al., 2020
	GW	WGBS	CpG sites in whole genome by NGS	Li et al., 2018
		MCTA-seq	Analysis of CpG islands by NGS	Liu X. et al., 2019
		TBS	Target CpG sites analysis by NGS	Liu M. C. et al., 2020
		cf-RRBS	Enzymatic digestion with MspI and NGS	De Koker et al., 2019
		Microarrays	~850.000 CpG sites	Gallardo-Gomez et al., 2018
		cfMeDIP-seq	Immunoprecipitation and NGS	Shen et al., 2018
Hydroxymethylation	GW	5hmC-Seal	Genomic 5hmC analysis by NGS	Li et al., 2017
Histone modifications	LS	ChiP	Chromatin immunoprecipitation and qPCR	Gezer et al., 2012
	GW	cfChIP-seq	Chromatin immunoprecipitation and NGS	Vad-Nielsen et al., 2020
Nucleosome positioning	GW	WGS	Analysis of DNA fragmentation by NGS	Mouliere et al., 2018
NcRNAs	LS	Nanostring	Panel of transcripts without amplification	Shukla et al., 2018
		RT-qPCR	Transcript detection by qPCR	Cojocneanu et al., 2020
		ddPCR	Transcript analysis by ddPCR	Gasparello et al., 2020
		Isothermic	Amplification at single temperature	Miao et al., 2016
		PNA-based biosensor	Transcript analysis without amplification	Metcalf et al., 2016
		ISH-LNA	ISH and LNA for transcript detection in CTCs	Ortega et al., 2015
		Droplet microfluidic	Multiple transcripts in CTCs with droplet microfluidic	Li et al., 2019
		Microarrays	Multiple transcripts by microarrays	Cojocneanu et al., 2020
	GW	RNA-seq	Transcriptomic analysis by NGS	Amorim et al., 2017

*LS, locus-specific; GW, genome-wide; MSP, methylation-specific PCR; qMSP, quantitative methylation-specific PCR; MS-HRM, methylation specific high resolution melting; scAEBS, single-cell agarose-embedded bisulfite sequencing; BEAMing, beads, emulsion, amplification, and magnetics dPCR, digital PCR; ddPCR, droplet digital PCR; MBD, methyl-CpG binding protein; WGBS, whole genome bisulfite sequencing; NGS, next-generation sequencing; MCTA-seq, methylated CpG tandem amplification and sequencing; TBS, targeted bisulfite sequencing; cf-RRBS, cell-free DNA reduced representation bisulfite sequencing; cfMeDIP-seq, cell-free methylated DNA immunoprecipitation and high-throughput sequencing; 5hmC, 5-hydroxymethylcytosine; ChiP, chromatin immunoprecipitation; cfChIP-seq, chromatin immunoprecipitation followed by sequencing of cell-free nucleosomes; WGS, whole genome sequencing; RT-qPCR, quantitative reverse transcription PCR; PNA, peptide nucleic acids; ISH-LNA, in situ hybridization-locked nucleic acid; RNA-seq, RNA sequencing*.

### DNA Modifications: Methylation and Hydroxymethylation

The detection of DNA methylation patterns is based on methods that (i) depend on sodium bisulfite conversion or (ii) are independent of sodium bisulfite, such as approaches based on immunoprecipitation and methyl-sensitive restriction enzymes (MSRE) (Huang and Wang, 2019). Methodologies that use bisulfite conversion are considered the gold standard for methylation analyses. These approaches are based on the principle that after sodium bisulfite treatment, methylcytosines do not undergo any change, whereas cytosine residues are converted into uracils (Frommer et al., 1992).

At the locus-specific scale, common bisulfite-based methods used in liquid biopsy include methylation-specific PCR (MSP), quantitative methylation-specific PCR (qMSP), MethyLight assay, methylation-sensitive high-resolution melting (MS-HRM), and more recently, digital PCR (dPCR)-based approaches (Li et al., 2009; Bao-Caamano et al., 2020). In particular, the detection of methylation by dPCR represents a quantitative and highly sensitive method that allows the analysis of very low amounts of DNA. Therefore, several dPCR-based methods, including methyl-BEAMing (Barault et al., 2015) and droplet digital PCR (ddPCR) (Boeckx et al., 2018), have been used for methylation analysis in cfDNA. Regarding CTCs, the first studies describing DNA methylation alterations in these cells were reported using MSP (Chimonidou et al., 2011). Recently, a new method has been described for methylation analysis of CTCs, based on agarose-embedded bisulfite treatment (AEBS), allowing to analyze the DNA methylation status of multiple loci of single CTCs by multiplex PCR (multiplexed-scAEBS) (Pixberg et al., 2017).

Moreover, genome-wide bisulfite-based approaches based on NGS allow to evaluate the whole methylome in liquid biopsy. For instance, whole-genome bisulfite sequencing (WGBS) has proved useful for the inspection of the whole methylation landscape of not only cfDNA from cancer patients (Li et al., 2018), but also of single CTCs and CTC clusters (Gkountela et al., 2019). Although WGBS is highly informative, the high cost of this approach is a limitation for its general implementation in a clinical setting (Legendre et al., 2015; Zhang et al., 2020). Therefore, other high-throughput bisulfite-based approaches, not allowing to analyze the complete methylome but enabling to assay a great number of CpGs in a genome-wide scale, have been proposed. For instance, by MCTA-seq (methylated CpG tandem amplification and sequencing) it is possible to analyze the methylation status of CGIs in cfDNA (Liu et al., 2019). Similarly, CpG-targeted bisulfite sequencing methods have proved useful for the analysis of methylation in cfDNA. In this regard, Liu et al. introduced a novel approach by which, after bisulfite treatment of plasma cfDNA, regions of interest are pulled down and sequenced, and the results are analyzed in combination with machine learning (Liu M. C. et al., 2020). Recently, cfDNA reduced representation bisulfite sequencing (cf-RRBS) has been developed as an alternative genome-wide bisulfite sequencing method to analyze the cfDNA methylome (De Koker et al., 2019). This new method is based on the reduced representation bisulfite sequencing (RRBS) technology, which was first described by Meissner et al. (2005). In cf-RRBS, cfDNA is dephosphorylated prior to enzymatic digestion by the methylation-insensitive restriction enzyme MspI and sequencing. This approach represents a cost-effective method that allows methylation profiling of cfDNA in liquid biopsy (Van Paemel et al., 2020). In addition to NGS methods, DNA methylation can also be analyzed at the genome-wide level in liquid biopsy using methylation microarrays. This methodology implies the use of bisulfite-converted DNA and has been applied to the study of cfDNA and CTCs from cancer patients (Friedlander et al., 2014; Gallardo-Gomez et al., 2018). These genome-wide approaches based on NGS and microarrays are very promising, but have still been used in a small number of works. Therefore, more studies are necessary to validate their application for the analysis of epigenetic alterations in liquid biopsy.

One of the advantages of bisulfite conversion-based approaches is that they allow methylome profiling at base resolution (Sun et al., 2015). However, the use of bisulfite treatment for methylation analysis has some limitations, such as a potentially high degradation of DNA or an incomplete bisulfite conversion (Grunau et al., 2001; Rand et al., 2002). To overcome these limitations, Shen et al. recently developed a genome-wide method based on cell-free methylated DNA immunoprecipitation and high-throughput sequencing (cfMeDIP-seq) (Shen et al., 2018). In contrast to bisulfite single-base resolution technologies, cfMeDIP-seq is a region-based method that unravels the methylation status of genomic regions of at least 100 bp in length (Shen et al., 2019). This new bisulfite-free method was adapted from a previous methylated DNA immunoprecipitation (MeDIP) protocol based on the use of anti-methylcytosine antibodies (Taiwo et al., 2012). cfMeDIP-seq is a low-input (requiring 1 to 10 ng of DNA) and sensitive (with a detection limit down to 0.001%) approach that can be used for both early- and late-stage detection of multiple types of tumors (Shen et al., 2018, 2019). Recently, another enrichment method based on immunoprecipitation was developed to detect cfDNA methylation. This new locus-specific method is based on the immunoprecipitation of methyl-CpG binding (MBD) proteins coupled with ddPCR (MBD–ddPCR) (Shinjo et al., 2020). This highly sensitive technique allows the detection of methylation sites in cfDNA.

Similar to DNA methylation, genome-wide hydroxymethylation profiles can be obtained from cfDNA of cancer patients. This can be achieved by 5hmC-Seal, a robust and efficient sequencing method that has proved useful for detecting 5hmC in cfDNA with high sensitivity and specificity (Li et al., 2017).

### Histone Modifications and Nucleosome Positioning

The global distribution of specific PTMs of histones in cells can be assayed by chromatin immunoprecipitation with massively parallel DNA sequencing (ChIP-seq) (Barski et al., 2007). Recently, Sadeh et al. developed a method to perform ChIP-seq of cell-free nucleosomes (cfChIP-seq). This method enables the capture of circulating nucleosomes with different active chromatin marks that maintain the cell-of-origin genomic distribution of modifications and expression patterns (Sadeh et al., 2019). Other similar approaches have been used to quantify the level of histone marks associated with circulating cell-free nucleosomes in plasma of cancer patients (Vad-Nielsen et al., 2020). In addition to these high-throughput technologies, circulating nucleosomes can also be analyzed by chromatin immunoprecipitation (ChIP) followed by quantitative PCR to detect histone modifications in individual genes (Gezer et al., 2012). Other methods, such as enzyme-linked immunosorbent assay (ELISA), are used to quantify the occurrence of specific histone marks based on their levels in circulating nucleosomes (Rahier et al., 2017).

In addition, the fragmentation patterns of cfDNA depending on nucleosome positioning can be analyzed using genome-wide fragmentation methods, based on the combination of NGS and machine learning. These approaches require a very low input of cfDNA from different types of fluids, allowing even early detection of cancer (Mouliere et al., 2018). Other genome-wide fragmentation methods, such as DNA evaluation of fragments for early interception (DELFI), have been recently proposed (Cristiano et al., 2019). These kinds of fragmentation approaches are based on prior knowledge on the different lengths of cfDNA fragments originating from tumor and non-tumor cells (Jiang et al., 2015). Moreover, the landscape of cfDNA fragmentation is associated with nucleosome occupancy and epigenetic regulation (Ivanov et al., 2015). In turn, nucleosome occupancy of cfDNA correlates well with the nuclear architecture, gene structure, and gene expression observed in cells (Snyder et al., 2016), providing relevant information about nucleosome organization and the tissue of origin (Ivanov et al., 2015; Mouliere et al., 2018; Cristiano et al., 2019).

### Non-coding RNAs

Genome-wide expression analyses, based on microarrays or NGS technologies, enable the detection of a large number of ncRNAs in a high-throughput manner. Therefore, microarrays and NGS have been used in liquid biopsy for the detection of several types of ncRNAs, including miRNAs, lncRNAs, and circRNAs (Amorim et al., 2017; Cojocneanu et al., 2020; Gasparello et al., 2020). Although both methods allow a comprehensive analysis of ncRNA transcripts, NGS displays higher sensitivity than microarrays and does not require previous knowledge of the target transcripts (Hurd and Nelson, 2009; Wang et al., 2019). On the other hand, several methods and instruments are based on targeted detection of transcripts in liquid biopsy. This is the case of the NanoString nCounter, a platform that directly detects the expression levels of a wide panel of ncRNAs without any enzymatic amplification (Shukla et al., 2018). Other technologies, such as reverse transcription quantitative PCR (RT-qPCR) and ddPCR, despite requiring enzyme-assisted amplification, allow to quantify the expression levels of specific ncRNA transcripts in liquid biopsy with high sensitivity. Importantly, RT-qPCR and ddPCR are usually used as gold standard methods to validate the results obtained by genome-wide approaches (Sole et al., 2019; Cojocneanu et al., 2020; Gasparello et al., 2020). However, since these PCR-based methods require several cycles of temperature variations, isothermal amplification approaches, which use a single temperature, have been developed for ncRNA analysis in liquid biopsy (Miao et al., 2016). In contrast, other methods for miRNA detection, such as the use of peptide nucleic acids (PNAs)-based fluorogenic biosensors, do not require amplification (Metcalf et al., 2016). Some targeted approaches have also been shown to be specifically useful for the analysis of ncRNAs, especially miRNAs, in CTCs. Among these, *in situ* hybridization (ISH) with locked-nucleic-acid (LNA) probes has been successfully applied for the detection of miRNAs in CTCs (Ortega et al., 2015), since the use of LNA probes increases the efficiency of ISH for the detection of miRNAs (Kubota et al., 2006). Other methods for the analysis of CTCs have been recently developed, based on signal amplification in microfluidic droplets for single-cell analysis of multiple miRNAs (Li et al., 2019).

## Deregulation of Epigenetic Mechanisms in Colorectal Cancer and Liquid Biopsy

CRC originates from an accumulation of both genetic and epigenetic alterations in normal colon epithelial cells, leading to their transformation, first into adenomas and then into adenocarcinomas (Fearon and Vogelstein, 1990; Lao and Grady, 2011). Epigenetic alterations are involved in all the steps of the adenoma-carcinoma sequence, participating in the initiation, progression, and metastasis of CRC (Kim et al., 2006; Wendt et al., 2006; Silva-Fisher et al., 2020). Importantly, the disruption of the epigenetic machinery has been proposed as a hallmark of cancer by several authors (Esteller, 2007; Bates, 2020).

Promoter hypermethylation of single TSGs has been widely described in colorectal tumor cells. In particular, the hypermethylation of CGIs in promoters of relevant genes (*p16/CDKN2A, MLH1, MGMT, APC*, and *TIMP3*, among others) is associated with CRC pathogenesis (Gonzalez-Zulueta et al., 1995; Hiltunen et al., 1997; Kane et al., 1997; Esteller et al., 1999; Lee et al., 2004). In addition, the hypermethylation of specific groups of genes has shown implications for this disease. In this sense, Toyota et al. described for the first time the CpG island methylator phenotype (CIMP), characterized by the hypermethylation of CGIs in the promoter regions of a panel of TSGs from a subset of CRC and adenoma tissues (Toyota et al., 1999). CIMP-High (CIMP-H) is considered a molecular subtype of sporadic CRC characterized by a high degree of methylation in CIMP-specific loci. Most studies have defined CIMP using a classic methylated-in-tumor (MINT) marker panel: *p16/CDKN2A, MLH1, MINT1, MINT2*, and *MINT31* (Nazemalhosseini Mojarad et al., 2013). However, subsequent studies have used other types and number of genes to define CIMP, thereby increasing the difficulty of its implementation in the clinic (Weisenberger et al., 2006; Ogino et al., 2007). On the other hand, recent advances in the characterization of genome-wide DNA methylation patterns have allowed the discovery of methylation signatures based on multiple CpG sites associated with CRC (Sandoval et al., 2011; Luo et al., 2014). This type of epigenomic methodology has revealed that the disruption of methylation in colorectal tumor cells can affect both coding and non-coding genes, such as those producing miRNAs and lncRNAs (Mori et al., 2011; Diaz-Lagares et al., 2016a). In addition to the hypermethylation of TSGs, colorectal tumors are also characterized by global hypomethylation, presenting a low methylation status in LINE-1 repetitive sequences (Suter et al., 2004). On the other hand, total 5hmC levels are reduced in colon tumor cells compared to normal cells (Gilat et al., 2017). However, tumor cells can also show high levels of 5hmC in transcriptionally active regions, associated with the expression of particular coding and non-coding genes; this phenomenon contributes to CRC pathogenesis, even in early stages (Hu et al., 2017; Gao et al., 2019).

Colorectal tumors are also characterized by the disruption of histone modification patterns, such as methylation [e.g., methylation of lysine 9 on H3 (H3K9me)] and acetylation [e.g., acetylation of lysine 27 on H3 (H3K27ac)] (Liu et al., 2013; Karczmarski et al., 2014). In addition, the positioning of nucleosomes can also be modified in tumors, and such alteration has implications for the reorganization of chromatin and gene expression (Hesson et al., 2014). In summary, the positioning of nucleosomes over the TSS and differences in the occurrence of histone epigenetic marks can regulate gene expression in colorectal tumor cells (Gimeno-Valiente et al., 2019).

The expression of several types of ncRNAs, including miRNAs and lncRNAs, is usually dysregulated in CRC. For instance, the overexpression of particular miRNAs, such as miR-21, is associated with the activation of relevant specific pathways that promote pathogenesis of colorectal tumor cells (Xiong et al., 2013). In addition, recent transcriptomic analyses have revealed CRC-specific expression signatures of miRNAs, lncRNAs, and other types of ncRNAs (Di et al., 2020; Li et al., 2020; Song et al., 2020). One of the epigenetic mechanisms that may control the expression levels of these ncRNAs in colorectal tumor cells is DNA methylation. For instance, this is the case of the lncRNA TP53TG1, which exerts tumor suppressor activity in gastrointestinal tumors and participates in the p53 response to DNA damage (Diaz-Lagares et al., 2016a).

Of note, advances in our understanding of aberrant epigenetics have led to the identification of epigenetic alterations in liquid biopsy elements (cNAs, CTCs, and cEVs) associated with all steps of CRC progression (Gasch et al., 2015; Zeng et al., 2018; Jung et al., 2020). Disruption of epigenetic mechanisms has great relevance in cancer dissemination and metastasis of solid tumors, which remains the leading cause of cancer-related death (Gupta and Massague, 2006). For example, hypermethylation of TSGs (e.g., *CDKN2A*), in both tumor tissue and cfDNA, is broadly associated with distant metastasis in CRC patients (Mitomi et al., 2010). In addition, hypomethylation of LINE-1 in plasma cfDNA of CRC patients is also correlated with disease progression; this association is stronger in tumors with higher size, lymph node affectation, and distant metastasis (Nagai et al., 2017). More recently, the epigenetic markers of CTCs have been explored to better understand cancer progression (Chimonidou et al., 2011). In particular, Lyberopoulou et al. were the first to determine the DNA methylation profile of cancer-related genes in CTCs of CRC patients. This study revealed that CTCs from CRC patients are characterized by hypermethylation of the *SFRP2* promoter and exon 1 of *VIM* (Lyberopoulou et al., 2017), which are genes related to epithelial-to-mesenchymal transition (EMT) and CRC metastasis (Shirahata et al., 2009; Loboda et al., 2011; Vincent and Postovit, 2017). In recent years, the analysis of single CTCs with genome-wide methylation (Gkountela et al., 2019) or multiplex approaches (Pixberg et al., 2017) has provided relevant information on cancer dissemination. However, the study of epigenetic alterations in CTCs from CRC patients remains limited.

In addition to CTCs, cancer-derived EVs play a major role in cancer progression as they participate in pre-metastatic niche formation (de la Fuente et al., 2015; Becker et al., 2016). For example, it has been shown that exosomal miR-25-3p from CRC cells regulates the expression of target genes, promoting vascular permeability, angiogenesis, and the formation of liver and lung metastasis in preclinical models (Zeng et al., 2018). Of note, plasma exosomal miR-25-3p levels are significantly higher in metastasic CRC patients than in those with no metastases, indicating a role for this miRNA in pre-metastatic niche formation (Zeng et al., 2018). On the other hand, the fact that serum exosomal miR-375 is less abundant in CRC patients with liver metastasis, together with functional studies, suggests its role as a tumor suppressor through the inhibition of the Bcl-2 pathway (Zaharie et al., 2015). Furthermore, recent results have indicated that decreased levels of serum exosomal miR-638 (Yan et al., 2017) and miR-548c-5p (Peng et al., 2018) are associated with liver metastasis in CRC patients.

Considering the relevance of epigenetic alterations in cancer dissemination and progression, there is a great interest in the development of epigenetic modifiers that can function as epidrugs. Importantly, some of these epigenetic modifiers have been tested preclinically or in early-phase clinical trials for CRC, representing a promising field for the treatment of this tumor (Baretti and Azad, 2018).

## Clinical Utility of Epigenetic Marks in Liquid Biopsy as CRC Biomarkers

**Figure 2:**
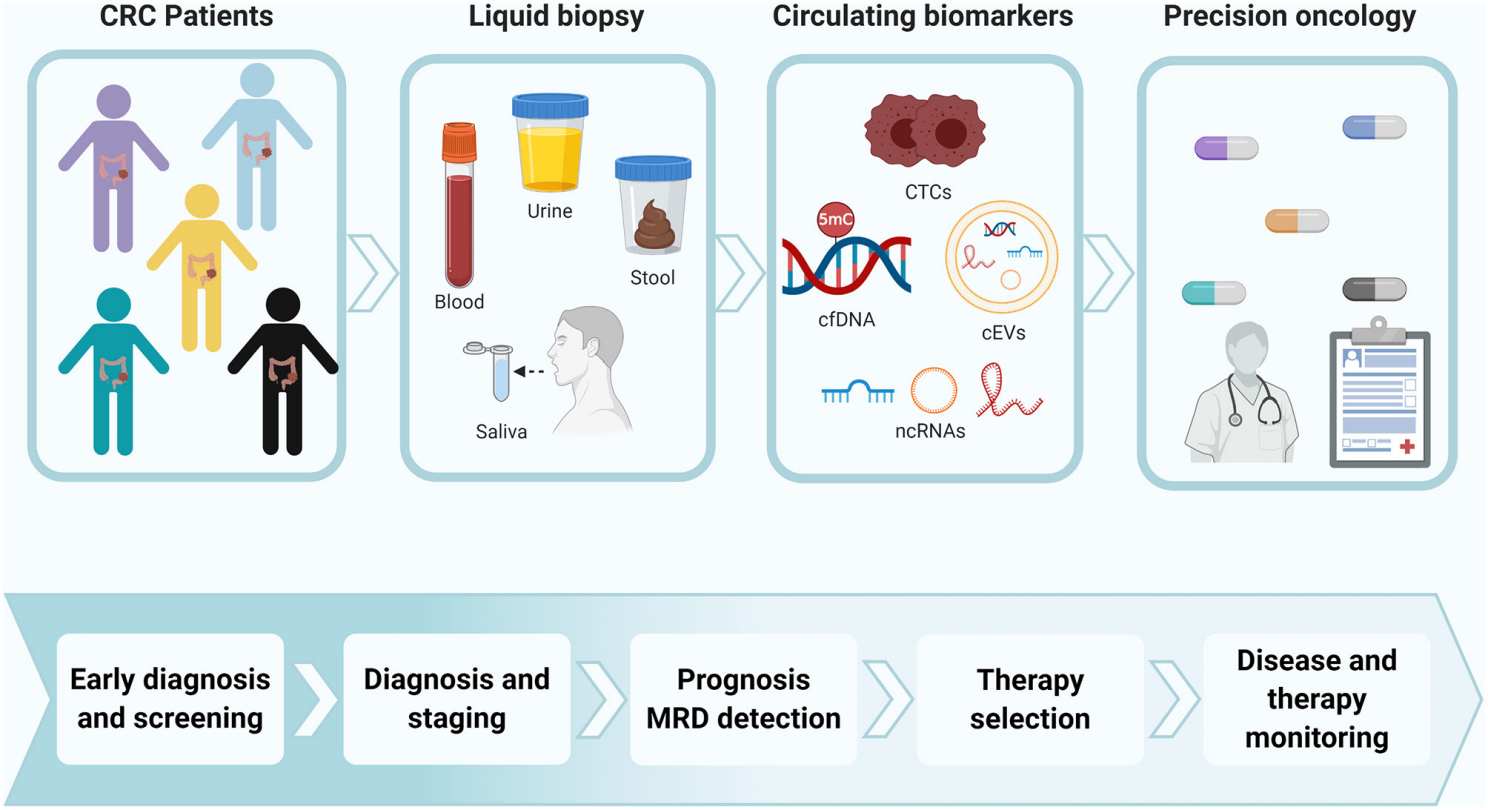
Epigenetic biomarkers in liquid biopsy for precision oncology of CRC patients. Colorectal primary tumors and metastasis can release epigenetic biomarkers into different types of biological fluids. The disruption of these epigenetic mechanisms can be detected in circulating tumor cells (CTCs), nucleic acids (cNAs) and extracellular vesicles (cEVs), showing clinical relevance as therapeutic targets and tumor biomarkers for early detection, prognosis, monitoring, therapy selection, and evaluation of therapeutic response in CRC. The detection of these epigenetic biomarkers in liquid biopsy have a great value to personalize the management of CRC patients. CRC, colorectal cancer; CTCs, circulating tumor cells; cfDNA, cell-free DNA; ncRNAs, non-coding RNAs; MRD, minimal residual disease. Created with BioRender.com.

Alterations in epigenetic marks have shown great utility in both tissue and liquid biopsies as tumor biomarkers for early detection, prognosis, monitoring, and evaluation of therapeutic response in CRC (Jung et al., 2020). Of these epigenetic alterations, changes in DNA methylation patterns and ncRNA expression are among the most well-known epigenetic biomarkers of CRC in liquid biopsy. Epigenetic biomarkers have been detected in all the components of liquid biopsy as well as in multiple biological fluids (Figure 2). Although blood is the most explored biological fluid for the study of these types of biomarkers, other fluids (such as stool and saliva) have also been considered as a relevant source of epigenetic tumor biomarkers for CRC. Tables 2–**7** include examples of representative circulating epigenetic biomarkers analyzed in blood and other fluids associated with clinical applications for CRC patients.

**Table 2:** DNA modifications in cfDNA of blood as epigenetic biomarkers of CRC.

**Feature**	**Biomarker/Assay**	**Clinical application**	**References**
Methylation	Epi proColon (*SEPT9*)	Diagnosis	Lofton-Day et al., 2008
	ColoDefense (*SEPT9, SDC2)*	Diagnosis	Zhao G. et al., 2019
	*SFRP1, SFRP2, SDC2, PRIMA1*	Diagnosis	Bartak et al., 2017
	SpecColon (*SFRP2, SDC2*)	Diagnosis	Zhao et al., 2020b
	TriMeth (*C9orf50, KCNQ5, CLIP4*)	Diagnosis	Jensen et al., 2019
	Methylation of multiple CpG sites	Diagnosis	Liu M. C. et al., 2020
	Methylation of genomic regions	Diagnosis	Shen et al., 2018
	*HPP1, HLTF*	Prognosis	Wallner et al., 2006
	*RARB, RASSF1A*	Prognosis	Rasmussen et al., 2018
	*EYA4, GRIA4, ITGA4, MAP3K14-AS1, MSC*	Monitoring	Barault et al., 2018
	*SEPT9, DCC, BOLL, SFRP2*	Monitoring	Bhangu et al., 2018
	*WIF1, NPY*	Monitoring	Garrigou et al., 2016
	*BCAT1, IKZF1*	Monitoring	Young et al., 2016
Hydroxy-methylation	5hmC	Diagnosis/Prognosis	Gao et al., 2019

### Blood-Based Circulating Epigenetic Biomarkers

#### DNA Modifications: Methylation and Hydroxymethylation

A plethora of epigenetic biomarkers based on altered methylation have been evaluated in blood-derived cfDNA for the management of CRC (Table 2). Among them, plasma SEPTIN9 (*SEPT9*) methylation is one of the most studied epigenetic biomarkers for the screening and early detection of this tumor (Lofton-Day et al., 2008; Church et al., 2014). Epi proColon was the first commercially available test for the detection of plasma *SEPT9* methylation, and Epi proColon 2.0 constitutes the improved second generation of this test. This qualitative assay is based on the detection of methylation in the promoter region of the *SEPT9* gene from plasma cfDNA by real-time PCR. Importantly, the Epi proColon test was the first blood-based assay approved by the FDA for the screening of CRC (Pickhardt, 2016; Issa and Noureddine, 2017). According to several studies, assessing the promoter methylation status of *SEPT9* allows to differentiate between CRC patients and healthy individuals with high overall sensitivity and specificity (Wang et al., 2018), although this assay has a limited capacity to predict precancerous lesions or adenomas (Church et al., 2014). In addition to this limitation, there are other aspects that have hampered the clinical implementation of this assay, including the high heterogeneity of the analytical characteristics among studies and its poor cost effectiveness in comparison with other methods (Wang et al., 2018). Recently, the combined analysis of *SEPT9* and *SDC2* methylation levels in blood has led to the development of a new test (ColoDefense) allowing significantly improved detection of CRC and adenomas, and thus representing a promising tool for tumor screening and early detection (Zhao G. et al., 2019). Other studies have described how the combined analysis of the methylation profiles of several genes in plasma allows for discrimination between healthy controls and patients with adenomas or CRC. For example, the analysis of a gene panel including *SFRP1, SFRP2, SDC2*, and *PRIMA1* allowed to distinguish between CRC patients and healthy controls with high sensitivity and specificity (Bartak et al., 2017). Similarly, with the SpecColon test it was possible to analyze the methylation patterns of plasma *SFRP2* and *SDC2* simultaneously, thereby accurately detecting CRC and advanced adenomas (Zhao et al., 2020b). In another study, the analysis of methylation patterns in a three-gene panel (*C9orf50, KCNQ5*, and *CLIP4*) in plasma, through a test called TriMeth, enabled early detection of CRC with good sensitivity and specificity (Jensen et al., 2019).

Furthermore, approaches based on methylation microarrays and NGS have been used to identify epigenetic biomarkers in cfDNA for cancer detection. For instance, the analysis of ~850,000 CpGs in pooled cfDNA samples by MethylationEPIC array highlighted 1,384 differentially methylated CpG sites that discriminate CRC patients from healthy controls (Gallardo-Gomez et al., 2018). Moreover, the combination of NGS with machine learning has enabled the development of a test based on ~1 million CpG sites capable of detecting and localizing more than 50 tumor types, including CRC (Liu M. C. et al., 2020). Another research group designed a targeted NGS assay based on 9,223 hypermethylated CpG sites obtained from The Cancer Genome Atlas (TCGA), which proved useful for identifying advanced CRC as well as other tumor types (Liu et al., 2018). In addition, the PanSeer assay, which considers 10,613 CpG sites, allowed the detection of five cancer types, including CRC, regardless of the tissue of origin. Importantly, this assay enabled to detect the presence of cancer in asymptomatic individuals years before standard diagnosis (Chen et al., 2020). Another approach for the analysis of cfDNA methylation patterns, based on the combination of immunoprecipitation and NGS (cfMeDIP), was demonstrated to be effective for the detection of CRC and other tumor types (Shen et al., 2018).

At the prognosis level, several studies have revealed that the analysis of DNA methylation in liquid biopsy is useful to predict the outcome of patients. The potential prognostic biomarker for CRC that has been most studied is the hypermethylation of the *p16* promoter in blood, which has been associated with worse overall survival (Xing et al., 2013). Similarly, several studies have analyzed the methylation status of other genes, such as *HPP1* and *HLTF*, showing that hypermethylation of these genes in serum samples indicates worse prognosis and higher mortality of CRC patients (Wallner et al., 2006; Philipp et al., 2012). Another study demonstrated that the median number of hypermethylated promoter regions was higher in CRC patients with distant metastasis than in those without metastasis. These authors also found that the hypermethylation of *RARB* and *RASSF1A* was associated with the aggressiveness of the disease, representing an independent predictive factor of worse overall survival (Rasmussen et al., 2018).

Circulating DNA methylation can also be analyzed in series of fluid samples to monitor tumor burden and evaluate the therapeutic response of CRC patients to different types of treatments, including chemotherapy and anti-EGFR therapy (Barault et al., 2018; Bhangu et al., 2018). In this regard, the hypermethylation of two genes (*WIF1* and *NPY*) in cfDNA has been described as a surrogate biomarker of tumor burden and applied to monitor patients without the need for mutational analysis in liquid biopsy (Garrigou et al., 2016). Of note, these types of circulating epigenetic alterations correlate with tumor volume and recurrence better than classical biomarkers, such as carcinoembryonic antigen (CEA) and carbohydrate antigen (CA) 19-9 (Young et al., 2016; Bhangu et al., 2018; Symonds et al., 2020). In line with these results, the analysis of circulating methylation markers has also shown consistency with the output of imaging tests in the assessment of response to therapy and surgery (Boeckx et al., 2018). All these findings indicate the potential of the analysis of circulating DNA methylation for improving the clinical evaluation of CRC patients and, if necessary, promptly redefining the treatment strategy.

The analysis of DNA methylation has also been explored in CTCs isolated from blood, although the number of studies focused on this circulating tumor population is still very low. Interestingly, when comparing the methylation levels of *VIM* and *SFRP2* in CTCs of CRC patients with those of tumor tissues, researchers found a strong correlation between the methylation status of the *SFRP2* promoter in CTCs and that of the corresponding tissue, but a weaker correlation in the case of *VIM*. Therefore, although the CTC population can show a different methylation pattern than tumor cells located in primary tissue and metastasis due to the gain of specific characteristics in the bloodstream, these data demonstrated that methylation profiling of CTCs in CRC patients represents a promising non-invasive approach for tumor detection (Lyberopoulou et al., 2017).

In addition to methylation, differential hydroxymethylation patterns of plasma cfDNA have been observed between patients with CRC and healthy controls, suggesting that markers of this epigenetic modification could also be used as a non-invasive tool for early detection and prognosis in CRC (Table 2). Importantly, the efficiency by which the presence of 5hmC in cfDNA points at CRC was shown to be similar to that in tumor tissues, and higher than that of well-known biomarkers, such as CEA, CA 19-9, and methylated *SEPT9* (Li et al., 2017).

#### Histones and Nucleosomes

The study of histone modifications in blood-circulating nucleosomes (Table 3) has revealed that they can also contribute to CRC detection. For example, low levels of the circulating histone marks H3K9me3, H4K20me3, and H3K27me3 have been proposed as biomarkers for the diagnosis of CRC (Gezer et al., 2013, 2015). The combination of different PTMs detected in circulating nucleosomes has also proved useful for CRC screening (Rahier et al., 2017). In addition, high concentrations of circulating nucleosomes in CRC patients have been associated with disease progression, poor therapy response, and reduced survival (Fahmueller et al., 2012). Notably, the levels of nucleosomes in cancer patients are dynamic and thus can be useful to indicate the response to therapy in real time (Holdenrieder et al., 2001).

**Table 3:** Histone/nucleosomes in blood as epigenetic biomarkers of CRC.

**Feature**	**Biomarker/Assay**	**Clinical application**	**References**
Histone modifications	H3K9me3, H4K20me3, H3K27me3	Diagnosis	Gezer et al., 2013, 2015
	H2AK119Ub, H3K9Ac, H3K27Ac	Diagnosis	Rahier et al., 2017
	H2AK119Ub, H3K9Ac, H4K20me3	Diagnosis	Rahier et al., 2017
Nucleosomes	Concentration levels	Prognosis	Fahmueller et al., 2012
	Concentration levels	Monitoring	Holdenrieder et al., 2001
	DNA fragmentation	Diagnosis	Cristiano et al., 2019
	DNA fragmentation	Therapy response	Kitahara et al., 2016

Nucleosome occupancy is closely related to the fragmentation patterns of cfDNA (Ivanov et al., 2015). Notably, Mouliere et al. found that in multiple cancer types, including CRC, plasma cfDNA fragments exhibited different sizes between healthy individuals and cancer patients (Mouliere et al., 2018). Therefore, these authors proposed cfDNA fragmentation patterns as epigenetic biomarkers for early cancer detection. Consistently, fragmentation profiling of plasma cfDNA proved effective for CRC diagnosis, even when the tumor origin was initially unknown (Cristiano et al., 2019). The level of cfDNA fragmentation can also be associated with the prognosis of CRC patients, as patients displaying higher fragmentation showed worse prognosis (El Messaoudi et al., 2016). In addition, the analysis of cfDNA fragmentation patterns has proved useful for early detection of MRD after surgery and to predict the response of CRC patients to immunochemotherapy (Table 3).

#### Non-coding RNAs

Similar to other epigenetic alterations, the presence of ncRNAs in blood is a relevant source of biomarkers for CRC management (Table 4). For instance, a recent meta-analysis revealed that circulating miR-21 is a promising biomarker for CRC detection, and that its diagnostic properties can be improved by combining it with other biomarkers (Peng et al., 2017). Moreover, numerous studies have identified circulating miRNA signatures and applied them for non-invasive early detection of CRC; this enabled to distinguish healthy controls, patients with precancerous lesions (advanced adenomas), and CRC patients with high sensitivity and specificity (Wang et al., 2012; Kanaan et al., 2013; Herreros-Villanueva et al., 2019). The analysis of miRNAs in blood can also provide prognostic information. For instance, increased levels of circulating miR-210 and miR-141 are associated with shorter survival rates (Cheng et al., 2011; Wang et al., 2017). In contrast, high plasma levels of miR-23b are associated with longer survival (Kou et al., 2016). The levels of different miRNAs in blood can also provide information about the patient's disease stage (Sun et al., 2016), and represent a valuable tool for early detection of recurrence after surgery (Yuan et al., 2017) and the evaluation of response to therapy in CRC patients (Hansen et al., 2015; Schirripa et al., 2019).

**Table 4:** Non-coding cfRNAs in blood as epigenetic biomarkers of CRC.

**Feature**	**Biomarker/Assay**	**Clinical application**	**References**
MiRNAs	miR-21	Diagnosis	Peng et al., 2017
	miR-601, miR-760	Diagnosis	Wang et al., 2012
	miR-532-3p, miR-331, miR-195, miR-17, miR-142-3p, miR-15b, miR-532, miR-652	Diagnosis	Kanaan et al., 2013
	miR-431, miR-15b, miR-139-3p	Diagnosis	Kanaan et al., 2013
	miR-19a, miR-19b, miR-15b, miR-29a, miR-335, miR-18a	Diagnosis	Herreros-Villanueva et al., 2019
	miR-210	Prognosis	Wang et al., 2017
	miR-141	Prognosis	Cheng et al., 2011
	miR-23b	Prognosis	Kou et al., 2016
	miR-96, miR-203, miR-141, miR-200b	Prognosis	Sun et al., 2016
	miR-29a, miR-200b, miR-203, miR-31	Prognosis	Yuan et al., 2017
	miR-31, miR-141, miR-16	Monitoring	Yuan et al., 2017
	miR-126	Therapy response	Hansen et al., 2015
	miR-21, miR-221, miR-760	Therapy response	Schirripa et al., 2019)
LncRNAs	HOTAIR, CCAT1	Diagnosis	Zhao et al., 2015
	91H, PVT-1, MEG3	Diagnosis	Liu et al., 2019
	XLOC_006844, LOC152578, XLOC_000303	Diagnosis	Shi et al., 2015
	SNHG11	Diagnosis	Xu et al., 2020
	HOTAIR	Diagnosis/Prognosis	Svoboda et al., 2014
CircRNAs	circ-CCDC66, circ-ABCC1, circ-STIL	Diagnosis	Lin et al., 2019
	hsa_circ_0082182, hsa_circ_0000370, hsa_circ_0035445	Diagnosis	Ye et al., 2019
	hsa_circ_0007534	Diagnosis/Prognosis	Zhang et al., 2018
	hsa_circ_0002320	Prognosis	Yang N. et al., 2020

Other ncRNAs, such as lncRNAs, are also deregulated in CRC and can provide relevant clinical information (Table 4). For example, elevated plasma levels of the lncRNA HOTAIR, alone or in combination with other lncRNAs, have proved useful not only for CRC screening, but also as a biomarker associated with worse prognosis and higher mortality (Svoboda et al., 2014; Zhao et al., 2015). Of note, other studies have proposed different combinations of circulating lncRNAs as diagnostic biomarkers for the detection of CRC and precancerous lesions (Shi et al., 2015; Liu et al., 2019; Xu et al., 2020). In addition to lncRNAs, the analysis of circRNAs in blood, as biomarkers with relevant clinical properties for CRC diagnosis and prognosis, has raised considerable interest (Lin et al., 2019; Yang N. et al., 2020).

Finally, ncRNA levels have also been analyzed in cEVs, especially in exosomes, of CRC patients (Table 5). Exosomal miRNAs (e.g., miR-21 and miR-139-3p) can be more or less abundant than normal in the blood of CRC patients and their analysis represents a valuable tool for CRC diagnosis and prognosis (Fu et al., 2018; Liu W. et al., 2020). Furthermore, the analysis of miRNAs in circulating exosomes has revealed important strategies for the identification of treatment-resistant patients (Jin et al., 2019). For example, the exosomal levels of the lncRNAs HOTTIP, LINC02418, and RPPH1 were found deregulated in CRC and thus proposed as potential markers for the diagnosis of CRC, as well as in other clinical contexts (Liang et al., 2019; Oehme et al., 2019; Zhao Y. et al., 2019). Finally, recent studies have focused on exosomal circRNAs (e.g., circ-133 and circ-PNN), suggesting a relevant role of these ncRNAs as biomarkers for CRC (Xie Y. et al., 2020; Yang et al., 2020).

**Table 5:** Non-coding RNAs in EVs of blood as epigenetic biomarkers of CRC.

**Feature**	**Biomarker/Assay**	**Clinical application**	**References**
MiRNAs	miR-19a, miR-20a, miR-143, miR-145, miR-150, let-7a	Diagnosis	Maminezhad et al., 2020
	miR-139-3p	Diagnosis/Metastasis monitoring	Liu W. et al., 2020
	miR-17-5p, miR-92a-3p	Prognosis	Fu et al., 2018
	miR-19a	Prognosis	Matsumura et al., 2015
	miR-21-5p, miR-1246, miR-1229-5p, miR-96-5p	Therapy response	Jin et al., 2019
LncRNAs	LINC02418	Diagnosis	Zhao Y. et al., 2019
	RPPH1	Diagnosis	Liang et al., 2019
	HOTTIP	Prognosis	Oehme et al., 2019
CircRNAs	circ-PNN	Diagnosis	Xie Y. et al., 2020

### Circulating Epigenetic Biomarkers in Alternative Fluids

#### DNA Modifications: Methylation and Hydroxymethylation

Numerous studies have reported the application of DNA methylation patterning in stool (Table 6) for the detection of CRC or precancerous lesions (Raut et al., 2020). In particular, methylation of the promoter region of *FBN1, SDC2*, or *VIM* has been reported as a biomarker for early CRC diagnosis (Chen et al., 2005; Guo et al., 2013; Han et al., 2019). In addition, the hypermethylation of a three-gene panel (*HPP1, SFRP2*, and *MGMT*) in stool showed clinical value for detecting CRC and precancerous colorectal lesions (Huang et al., 2007). Other studies have also highlighted the hypermethylation of the promoters of numerous genes in stool samples from patients affected by CRC or adenomas, such as *HIC1, COL4A1, COL4A2, GATA4, ITGA4, OSMR*, and *TLX2* (Lenhard et al., 2005; Kim et al., 2009; Liu et al., 2020). In addition, the methylation status of various miRNAs (e.g., miR-34a and miR-34b/c) was found to be altered in stool of CRC patients, and studied as a source of potential biomarkers (Kalimutho et al., 2011; Wu et al., 2014).

**Table 6:** DNA modifications in non-blood fluids as epigenetic biomarkers of CRC.

**Feature**	**Sample**	**Biomarker/Assay**	**Clinical application**	**References**
Methylation	Stool	*FBN1*	Diagnosis	Guo et al., 2013
	Stool	*SDC2, VIM*	Diagnosis	Chen et al., 2005
	Stool	*HPP1, SFRP2, MGMT*	Diagnosis/Screening	Huang et al., 2007
	Stool	miR-34a, miR-34b/c	Diagnosis	Kalimutho et al., 2011; Wu et al., 2014
	Stool	ColoGuard (*NDRG4, BMP3*)	Diagnosis	Imperiale et al., 2014
	Stool	ColoSure (*VIM*)	Diagnosis	Ned et al., 2011
	Stool	ColoDefense (*SEPT9, SDC2*)	Diagnosis	Zhao et al., 2020a
	Urine	*VIM*	Diagnosis/Screening	Song et al., 2012
	Urine	*NDRG4*	Diagnosis	Xiao et al., 2015
	Urine	*WIF1*	Diagnosis	Amiot et al., 2014
	Bowel lavage	mir-34b/c	Diagnosis	Kamimae et al., 2011
	Bowel lavage	miR-124-3, LOC386758, *SFRP1*	Diagnosis	Harada et al., 2014
Hydroxymethylation	Urine	5-hydroxymethylated cytosine nucleosides	Diagnosis	Guo et al., 2018

Importantly, some of the methylated biomarkers detected at an increased concentration in stool samples from CRC patients have been translated into clinical tests. This is the case of Cologuard, which is the first multi-target stool panel approved by the FDA for CRC screening (Pickhardt, 2016). The Cologuard assay combines the analysis of *NDRG4* and *BMP3* methylation, *KRAS* mutation, β-actin levels, and hemoglobin levels by immunochemistry. This multiparameter stool assay displays a high sensitivity in CRC detection, higher than that of FIT; however, Cologuard yields lower specificity in the case of asymptomatic patients, which represents a limit for its clinical implementation (Imperiale et al., 2014). Another similar FDA-approved test, ColoSure, allows to analyze *VIM* methylation in stool samples to identify the presence of precancerous adenomas or malignant colorectal tumors (Ned et al., 2011). Another strategy for addressing the methylation of known promoters is the application of the ColoDefense test to stool samples, which enables to detect the methylation status of *SEPT9* and *SDC2* with high sensitivity and specificity (Zhao et al., 2020a).

Similar to other biofluids, urine also contains cfDNA suitable for epigenetic analyses (Table 6). For instance, the *VIM* promoter in cfDNA from urine samples was found to display higher methylation in CRC patients than in controls, and thus methylation profiling of this promoter was proposed as a useful test for CRC screening (Song et al., 2012). The methylation status of *NDRG4* has also been explored in urine from CRC patients, showing higher diagnostic properties than that in blood and stool (Xiao et al., 2015). The easier manipulation of urine samples with respect to stool supports the value of this approach as a potential clinical method for CRC detection. The methylation levels of *WIF1, ALX4*, and *VIM* were also assessed in either urine or serum samples of CRC patients and controls with promising results for CRC diagnosis in the case of *WIF1* (Amiot et al., 2014). In addition, several epigenetic markers, including aberrant levels of methylated and hydroxymethylated cytosine nucleosides, have been analyzed in urine samples with promising results, and may be applied as potential biomarkers of CRC (Guo et al., 2018).

Interestingly, there are also other types of fluids that can be obtained from CRC patients. For example, mucosal wash fluid, in which DNA methylation is also detectable, can be collected during colonoscopy (Kamimae et al., 2011). Moreover, the release of cancer cells by the most aggressive tumors might impact the levels of methylated biomarkers in wash samples. To verify this hypothesis, the methylation levels of 15 genes were assessed in bowel lavage fluid (BLF) samples from a large cohort of individuals with CRC, adenomas, and small polyps, as well as from healthy individuals. The methylation levels of three gene promoters (*mir-124-3, LOC386758*, and *SFRP1*) showed good correlation with CRC, confirming that methylation studies in BLF samples represent a source of potential biomarkers for CRC detection (Table 6) (Harada et al., 2014).

#### Histones and Nucleosomes

As previously mentioned, nucleosome occupancy is closely related to the fragmentation patterns of cfDNA (Ivanov et al., 2015). In this regard, the different length of the DNA fragments present in stool has also been proposed as a good tool to discriminate CRC patients from healthy donors. Within this line of research, the integrity of stool DNA from CRC patients was analyzed using an oligonucleotide-based hybrid capture strategy to quantify DNA fragments of 200, 400, 800 bp, 1.3, 1.8, and 24 kb, unraveling the association between high-molecular-weight fragments and this tumor type (Boynton et al., 2003). The presence of long DNA fragments was also found to be increased in CRC patients with respect to healthy controls through the use of fluorescent primers and capillary electrophoresis (Calistri et al., 2004). Such presence of long DNA fragments in stool samples has been reported in several studies as a potential biomarker, either alone (Zou et al., 2006) or in combination with the presence of oncogenic mutations in different genes (*KRAS, APC*, or *p53*) (Ahlquist et al., 2000) or with altered methylation patterns (Ahlquist et al., 2012).

#### Non-coding RNAs

NcRNAs (mainly miRNAs) are stable in stool samples, and thus represent a relevant source of non-coding biomarkers (Table 7). For instance, the analysis of miR-451a and miR-144-5p levels enables to differentiate between patients with CRC and healthy donors with high sensitivity and specificity (Wu et al., 2017), while that of miR-20a levels is more suitable to discriminate CRC patients from both adenoma patients and healthy controls (Yau et al., 2016). In addition, the combined investigation of miR-421 and miR-27a-3p levels with hemoglobin quantification in stool was described as a valuable tool to improve the sensitivity of current screening strategies (Duran-Sanchon et al., 2020). Similarly, increased accumulation of several miRNAs (e.g., miR-21) has also been detected in stool from CRC patients with respect to that from healthy individuals or adenoma patients, and has thus been proposed as a potential screening tool (Wu et al., 2012).

**Table 7:** Non-coding cfRNAs in non-blood fluids as epigenetic biomarkers of CRC.

**Feature**	**Sample**	**Biomarker/Assay**	**Clinical application**	**References**
MiRNAs	Stool	miR-451a, miR-144-5p	Diagnosis	Wu et al., 2017
	Stool	miR-20a	Diagnosis	Wu et al., 2017
	Stool	miR-421, miR-27a-3p	Diagnosis	Duran-Sanchon et al., 2020
	Stool	miR-21	Diagnosis	Wu et al., 2012
	Saliva	miR-21	Diagnosis	Sazanov et al., 2017
	Saliva	miR-186-5p, miR-29a-3p, miR-29c-3p, miR-766-3p, and miR-491-5p	Diagnosis/Prognosis	Rapado-Gonzalez et al., 2019

On the other hand, several studies have demonstrated the potential of saliva as a source of biomarkers for non-oral cancer detection (Table 7). In particular, two studies have validated the feasibility of detecting increased levels of miRNAs in the saliva of CRC patients. In one of these studies, miR-21 levels were found to be increased in both plasma and saliva of patients with stage II–IV CRC compared to those of healthy individuals. Importantly, the sensitivity and specificity of CRC identification were higher when analyzing saliva samples (Sazanov et al., 2017). On the other hand, Rapado-González et al. performed a unique massive profiling of miRNAs in saliva samples from both CRC patients and healthy donors, and found a total of 22 miRNAs whose accumulation was specifically altered in CRC patients. The levels of most of these miRNAs had been previously described as altered in tissue or plasma samples from CRC patients. Moreover, five of these altered miRNAs (miR-186-5p, miR-29a-3p, miR-29c-3p, miR-766-3p, and miR-491-5p) showed potential as diagnostic tools to detect CRC. Overall, these two studies demonstrated that salivary miRNA analysis represents a novel approach to detect cancer-associated epigenetic alterations with potential clinical value (Rapado-Gonzalez et al., 2019).

MiRNA content was also investigated in cEVs isolated from peritoneal lavage fluid and ascites of CRC patients and control individuals. Such global characterization of cEV-associated miRNAs provided a list of 210 miRNAs whose abundance is significantly altered in CRC patients, most of which were less abundant. From this altered pattern of miRNA accumulation, the authors could identify a 10-miRNA signature with clinical value for CRC detection (Roman-Canal et al., 2019).

### Circulating Epigenetic Biomarkers in Blood vs. Alternative Fluids

As we have already described, nowadays there are several body fluids that can serve as liquid biopsies to interrogate epigenetic biomarkers in CRC. Beyond the great value of blood samples as a source of tumor material, stool, and other alternative liquid biopsies showed great potential to analyze clinically relevant biomarkers. Although liquid biopsy has the advantage of being non-invasive and accessible, the diagnostic utility of the different epigenetic biomarkers can be conditioned by the type of body fluid and biomarker analyzed. For example, the detection of some epigenetic biomarkers can be more sensitive in stool than plasma samples. Thus, some studies compared the performance of methylation biomarkers in plasma and stool in parallel, finding relevant differences. This is the case of *SEPT9* methylation, which was evaluated in stool and plasma from patients with adenomas and malignant CRC tumors. Both strategies showed similar sensitivity and specificity for the detection of CRC at all stages, however, the methylation levels of *SEPT9* in stool showed higher sensitivity for detecting adenomas and early CRC tumors, indicating that the methylation analysis of this gene in stool can improve CRC screening (Liu Y. et al., 2020). The higher diagnostic accuracy observed in stool respect to blood samples could be explained due to biomarkers can be released directly from tumor cells to the intestinal lumen (Osborn and Ahlquist, 2005). Similar results were obtained for the methylation analysis of *VIM*. Although in advanced CRC this biomarker showed similar diagnostic utility in both types of samples, in early stages the methylation of *VIM* offered higher diagnostic accuracy in stool than in plasma (Chen et al., 2005; Itzkowitz et al., 2007; Li et al., 2009). On the other hand, in independent studies the levels of methylated *SFRP2* in stool and plasma showed similar results in terms of sensitivity and specificity to detect adenomas (Zhang et al., 2014; Bartak et al., 2017). MiRNA biomarkers can also show differences between stool and blood samples. Thus, although the analysis of miR-92a expression was described as a good diagnostic tool in both types of fluids, the sensitivity of miR-92a for adenomas detection was higher in blood than stool (Ng et al., 2009; Wu et al., 2012). In addition, miR-21 was found to have a similar value for discriminating CRC patients from advanced adenomas in blood and stool, but in stool samples the data variability was higher (Peng et al., 2017). In this sense, it is important to mention that stool samples represent a very heterogeneous biological material difficult to normalize among individuals for the quantitative analysis of miRNAs. Standardization of procedures from stool collection and amount of starting material to RNA extraction and detection methods, have been proposed for the detection of CRC with fecal miRNAs (Marcuello et al., 2019).

Similar to stool samples, urine represents a completely non-invasive body fluid that can be collected without pain or risk for the patient, making the sample very suitable for mass-screening of epigenetic biomarkers in CRC. This fluid has shown promising results to analyze the methylation of some genes, such as *NDRG4*, which provided higher diagnostic power to detect CRC than the same biomarker analyzed in blood and stool (Xiao et al., 2015). Additionally, saliva also has some advantages as a diagnostic tool in comparison with blood samples. Its collection is even less invasive than blood and without causing any discomfort for the patient. Recently, miR-21 expression was assessed in peripheral blood and saliva samples obtained from patients with CRC at different stages and healthy controls. Although miR-21 levels in both saliva and plasma, showed diagnostic value for CRC screening, the analysis of saliva demonstrated higher sensitivity and specificity than blood (Sazanov et al., 2017).

Overall, the selection of the most appropriated body fluid source to analyze the different tumor epigenetic marks will depend on the specific biomarker, the clinical context and the level of clinical validation, which is nowadays higher in blood and stool samples than in other alternative body fluids.

## Conclusions and Future Perspectives

The interest in epigenetic alterations associated with CRC development and progression as potential clinical biomarkers or therapeutic targets has increased significantly in recent years. In the present review, we provide an overview of the different epigenetic mechanisms that regulate key steps of CRC development. Because of the clear advantage of epigenetic analysis in liquid biopsies as a non-invasive method for the dynamic characterization of CRC, we also summarize the techniques currently applied to characterize epigenetic modifications in liquid biopsy, and describe the circulating epigenetic biomarkers identified in different body fluids and their clinical potential for the personalized management of CRC.

There is a wide range of epigenetic mechanisms that are altered in CRC, including DNA methylation and hydroxymethylation, nucleosome positioning, histone modifications, and the expression of different ncRNAs (miRNAs, lncRNAs, and circRNAs). All these alterations can be explored in different fluid samples such as blood, stool, urine, or saliva, and represent a valuable source of clinical biomarkers. In fact, as detailed in this review, the differential methylation status of several gene promoters in CRC constitutes the rationale of several commercialized screening tests, such as Cologuard or Epi ProColon. However, substantial optimization is required prior to the general implementation of circulating epigenetic markers in the clinic to guide the management of CRC patients.

Indeed, some technical factors clearly hamper the potential translation of circulating biomarkers into the clinical routine. For example, CTCs, cNAs, and cEVs released by tumor and metastases into body fluids are usually poorly concentrated with respect to circulating non-tumor elements. Therefore, the methodology employed for their detection must be very sensitive in both localized and advanced CRC. On the other hand, new NGS-based strategies, together with ddPCR-based approaches, display improved sensitivity for the detection of epigenetic signatures in cfDNA, opening new avenues for early diagnosis of CRC. However, these approaches usually require sophisticated laboratory equipment, and are too laborious and expensive to be generically implemented into the clinical routine. Epigenetic patterns are starting to be explored also in CTCs, but mainly with the aim of characterizing the specific signatures associated with the disseminative behavior of these tumor cells, due to the difficulty of isolating CTCs even in metastatic patients. Together with their sensitivity, the specificity of epigenetic biomarkers represents a critical factor affecting their clinical utility. In fact, poor specificity of screening tests would result in unnecessary invasive evaluations and undesired side effects.

Molecular intratumoral heterogeneity also represents a clinical and technical challenge for the translation of epigenetic biomarkers in the clinical setting. It is well-acknowledged that CRC tumors are composed of multiple tumor clones with different biological properties. This heterogeneity also has an impact on the variability of several epigenetic marks, with possible clinical implications. Therefore, technical improvement is required for the study of epigenetic changes in single CTCs, which will be of great value to achieve a better understanding of CRC biology.

Another important aspect is the need for standardized protocols for sample collection, processing, and storage to improve the reproducibility of studies. In particular, sample collection and pretreatment are key steps for methylation studies, and a source of heterogeneity (Pharo et al., 2016; Merker et al., 2018; Zavridou et al., 2018). Therefore, efforts should be made to harmonize pre-analytical and analytical protocols according to the epigenetic alteration, circulating element, or body fluid under investigation. Hopefully, this standardization will be possible in the near future thanks to the commitment of international initiatives and scientific societies.

A major consideration for the clinical translation of epigenetic biomarkers is the cost effectiveness of the analyses. Nowadays, most technologies consist in time-demanding, mainly NGS-based approaches, which include library preparation and bioinformatic interpretation. However, in the future, these strategies should be associated with more adjusted costs and user-friendly bioinformatic solutions. To this purpose, it would be convenient to stimulate the technological development and commercial interest of epigenetic-based tests, thereby creating a competitive environment that will lead to significant benefits in terms of pricing and effectiveness.

Of note, the scientific community have focused their attention on the great potential of epigenomic approaches in the analysis of liquid biopsy for diagnostic purposes. For instance, one of the emerging trends is the use of broad panels of methylated biomarkers in cfDNA for pan-cancer detection. Moreover, recent studies on cfDNA have revealed the importance of NGS-based approaches for the analysis of methylation signatures, nucleosome positioning, or fragmentation footprints, opening a new scenario for epigenetics in liquid biopsy (Snyder et al., 2016; Liu et al., 2018; Cristiano et al., 2019; Liu M. C. et al., 2020). In addition, there is great interest in increasing the knowledge of the epigenetic characteristics of CTCs and cEVs also in terms of other epigenetic layers, such as 5hmC levels, differential presence of various types of ncRNAs, and epitranscriptomic modifications.

The methylation analysis of *SEPT9* (Epi proColon test) was the first blood-based assay approved by the FDA (Pickhardt, 2016; Issa and Noureddine, 2017), opening great opportunities for the clinical applications of circulating epigenetic biomarkers. One of the most challenging goals to be addressed is the clinical validation of the numerous epigenetic biomarkers that have provided promising results but have not yet reached a routine use, in order to assess their prognostic value for the outcome of patients with CRC. Indeed, the translation of the results obtained with epigenetic biomarkers into diagnostic tools is still limited, probably due to the lack of standardization and the limited number of large independent cohorts that have been analyzed. Therefore, more clinical trials, including the study of the dynamics of these epigenetics biomarkers, are warranted to validate their impact in terms of survival benefit. Similarly, numerous studies have shown that epigenetic alterations can be reversed through pharmacological intervention. However, evidence supporting the benefit of epigenetic modifiers in patients with CRC is still faint (Rezapour et al., 2019).

In summary, epigenetic circulating biomarkers have demonstrated a great potential for diagnosis of CRC, as well as for monitoring the progression and therapy response of CRC patients in a non-invasive and dynamic way. However, although significant progress has been made in the clinical translation of these biomarkers in recent years, further research is required to overcome some technologic and clinical barriers. Indeed, the translation of circulating epigenetic biomarkers into the clinical setting will require large multicenter studies to demonstrate the clinical benefit of their use. Such studies should be carried out in the near future and are expected to yield valuable results, toward a more personalized management of CRC patients.

## Author Contributions

RL-L, LM-R, and AD-L designed the work. AR-C, NC-F, AB-C, LM-R, and AD-L wrote the original manuscript and prepared the tables. NC-F, LM-R, and AD-L designed all the figures. AR-C, NC-F, AB-C, LM-R, RL-L, and AD-L reviewed the manuscript. LM-R and AD-L supervised the work. All the authors contributed to the article and approved the submitted version.

## Conflict of Interest

RL-L has received honoraria for participation in Advisory Boards from Roche, AstraZeneca, Merck, MSD, Bayer, BMS, Novartis, Janssen, Lilly, Pfizer and Leo; travel, accommodations and expenses from Pharmamar, Roche, BMS and Pierre Fabre; research funding from Roche and Merck; and is co-founder and shareholder in Nasasbiotech, S.L., Mtrap Inc. The remaining authors declare that the research was conducted in the absence of any commercial or financial relationships that could be construed as a potential conflict of interest.
